# Role of Autophagy in HIV-1 and Drug Abuse-Mediated Neuroinflammaging

**DOI:** 10.3390/v15010044

**Published:** 2022-12-23

**Authors:** Susmita Sil, Annadurai Thangaraj, Abiola Oladapo, Guoku Hu, Naseer A Kutchy, Ke Liao, Shilpa Buch, Palsamy Periyasamy

**Affiliations:** 1 Department of Pharmacology and Experimental Neuroscience, University of Nebraska Medical Center, Omaha, NE 68198, USA; 2 Centre for Excellence in Nanobio Translational Research, Anna University, BIT Campus, Tiruchirappalli 620024, Tamil Nadu, India; 3 Department of Animal Sciences, School of Environmental and Biological Sciences, Rutgers, The State University of New Jersey, New Brunswick, NJ 08901, USA; 4 Cedars-Sinai Medical Center, Smidt Heart Institute, Los Angeles, CA 90048, USA

**Keywords:** HIV-1, drug abuse, autophagy, neuroinflammation, aging

## Abstract

Chronic low-grade inflammation remains an essential feature of HIV-1 infection under combined antiretroviral therapy (cART) and contributes to the accelerated cognitive defects and aging in HIV-1 infected populations, indicating cART limitations in suppressing viremia. Interestingly, ~50% of the HIV-1 infected population on cART that develops cognitive defects is complicated by drug abuse, involving the activation of cells in the central nervous system (CNS) and neurotoxin release, altogether leading to neuroinflammation. Neuroinflammation is the hallmark feature of many neurodegenerative disorders, including HIV-1-associated neurocognitive disorders (HAND). Impaired autophagy has been identified as one of the underlying mechanisms of HAND in treated HIV-1-infected people that also abuse drugs. Several lines of evidence suggest that autophagy regulates CNS cells’ responses and maintains cellular hemostasis. The impairment of autophagy is associated with low-grade chronic inflammation and immune senescence, a known characteristic of pathological aging. Therefore, autophagy impairment due to CNS cells, such as neurons, microglia, astrocytes, and pericytes exposure to HIV-1/HIV-1 proteins, cART, and drug abuse could have combined toxicity, resulting in increased neuroinflammation, which ultimately leads to accelerated aging, referred to as neuroinflammaging. In this review, we focus on the potential role of autophagy in the mechanism of neuroinflammaging in the context of HIV-1 and drug abuse.

## 1. Introduction

The invention of combined antiretroviral therapy (cART) as a pharmaceutical intervention has improved the average life expectancy of people living with HIV-1 (PLWH), which is comparable to healthy individuals [1,2,3]. This therapeutic intervention has transformed HIV-1/acquired immunodeficiency syndrome (AIDS; formerly considered a death sentence), with its short post-diagnosis average lifespan, into a manageable chronic disorder like diabetes mellitus [4]. Despite the promising prospects of cART, the quality of life offered by this therapy is threatened by the prevalence of mild cognitive decline and loss of memory, commonly referred to as HIV-1-associated neurocognitive disorders (HAND), which afflicts 30–60% of PLWH [1,2,3]. The presence of comorbidities, such as diabetes mellitus, cardiovascular disorders, atherosclerosis, and neurological decline, is prevalent, especially among aged PLWH [5]. According to the Centers for Disease Control and Prevention, the increasing number of individuals diagnosed with HIV-1 infection aged 50 and above doubled in 2009 (~33%) from 2001 (~17%) [4,5]. The expectation for an increased diagnostic rate within this age range has thus created an emergency public health challenge [4,5]. The emergence of physiological changes has been implicated as a factor that increases the risk for accompanying comorbidities in aged HIV-1-infected individuals. This complexity aggravates even PLWH on cART therapy [4]. The contributory role of the physiological alterations employed by HIV-1 infection, chronic effects of residual HIV-1 proteins in low penetrating tissues, pain management dependence, and cART treatment remain largely unknown. Although, the constant immune activation occurring as a result of chronic low-level inflammation observed in PLWH is likely to accelerate aging phenotypes among those individuals [6,7,8,9,10,11,12,13,14,15,16]. HIV-1 has been well-documented to impact the immune system and the brain, which can be observed in about 8–15% development of severe HIV-1-associated dementia, an incidence significantly reduced by cART [4]. The efficacy of cART in reducing viremia, and the increase in the average quality of life of the HIV-infected population, have not prevented the continued presence of chronic low-level inflammation associated with accelerated aging and neurodegeneration [4,5]. The neurotoxic impact of cART and its potential effects on premature aging and inflammation are well-characterized [17].

The comorbidity of substance abuse (cocaine, methamphetamine [meth], opiates), with respect to HAND, has further complicated matters for PLWH [5]. This drug abuse is an essential driving factor of HIV-1 infection worldwide, as both are linked and have been shown to promote premature aging [18,19]. Accumulating evidence points to abuse of drugs in approximately 30% of PLWH afflicted with HAND [20,21]. Furthermore, there are also implications of exacerbated neurological aging in these individuals abusing drugs [20,21]. Recent reports indicate that individuals, including patients on cART therapy, who abuse drugs present higher viral load accelerate HIV-1 progression and AIDS-related opportunistic infection [4]. The progression of HIV-1 infection in individuals who abuse drugs is exacerbated by their non-compliance to the prescribed cART, causing recurrent viral activation [22]. This accelerated HIV-1 progression is due to the accessibility of the blood–brain barrier to the virus, thereby triggering the activation of glial cells and neurotoxins released by drug abuse, leading to the mediation of neuroinflammation [23]. The continued presence of low-level inflammation, especially in the central nervous system (CNS), is the primary driver of accelerated aging in PLWH [4,5]. Recent reports have shown a direct linkage between neuroinflammation and the accelerated aging process based on enhanced activation of glial cells in the aged population [5]. This indicates that many mechanisms, such as upregulated endoplasmic reticulum (ER) stress, reactive oxygen species (ROS), dysregulated toll-like receptor signaling (TLR) [24,25,26], and NOD-like receptor proteins (NLRPs) inflammasome activation, have been implicated as part of the molecular details of HIV-1, drug abuse, and cART effects on the CNS [27]. In addition to their addictive effects, drugs of use such as cocaine, morphine, meth, and alcohol have also been reported to be involved in caspase-mediated cell death, mitochondrial dysfunction, activation of transcription factors like cAMP response element-binding protein, and early genes (c-fos and c-jun), neuronal injury, glial activation, and neuroinflammation [28,29,30,31,32,33,34,35,36,37,38,39]. Additionally, cellular dysfunction and neuroinflammation can result from the dysregulation of autophagy by HIV-1/HIV-1 proteins, drug abuse, and cART [5]. 

Autophagy is an evolutionarily conversed process characterized by cellular degradation and recycling intracellular macromolecules into their constituent subunits in the lysosome [40,41]. Three morphologically distinct primary types of autophagy (macroautophagy, microautophagy, and chaperone-mediated autophagy) exist, but they all culminate in the delivery of cargo to the lysosome for degradation and recycling [41]. Macroautophagy is characterized by the protrusion or invagination of the membranes of a lysosome to capture and sequester cargo and intact organelles, while microautophagy sequesters cargo away from the lysosome [41,42]. Chaperone-mediated autophagy differs from the two primary types of autophagy by using chaperones to identify cargo proteins that contain a particular pentapeptide motif, which is unfolded and individually translocated across lysosomal membranes [42]. Of the three, macroautophagy is the most studied, as it involves the de novo synthesis of double-membrane vesicles (termed autophagosomes) to sequester cargo proteins/organelles and eventually transport them across the lysosome for degradation [43]. Autophagy is a highly regulated and conserved process that involves multiple autophagy-related proteins, transcriptional modification, post-translational modulations, and complex protein–protein interactions [40]. Autophagy mechanisms involve the phosphorylation of Unc-51-like autophagy activating kinase (ULK1/2) by upstream kinase that regulates its preinitiation complex [5]. This leads to the activation of class III phosphoinositide 3-kinase (PI3K), an autophagy initiation complex via the ULK1/2 phosphorylation, and B-cell lymphoma 2 (Bcl-2) to beclin 1 disruptive binding. This activation generates phosphatidylinositol-3-phosphate at the nucleation site in isolated membranes, causing multiple protein binding to form autophagosomes in the elongation step. The formed autophagosome is further stabilized by other proteins, such as E1 ligase, E2 ligase, and E3 ligase complex [5]. The maturation involves the autophagosome fusion with lysosomes to form autolysosome, degrading sequestered contents and releasing them to the cytoplasm for recycling [40]. The maturation step is characterized by changes in the adaptor protein, p62 expression [5].

## 2. Interplay of Autophagy, Inflammation, and Aging–HIV and Drug Abuse

Autophagy is an inherent cellular defense mechanism that is highly regulated and involved in degrading cytoplasmic components using lysosomes. The autophagosome and lysosome play the central role in autophagy, in which the autophagosome, a double-membrane organelle, engulfs cellular components/debris and transports it to a lysosome to break down using lysosomal enzymes [44]. In presenting debris to a lysosome, an autophagosome is not selective—it engulfs cellular organelles, protein aggregates, damaged proteins, and disease-causing pathogens [45].

**Figure 1:**
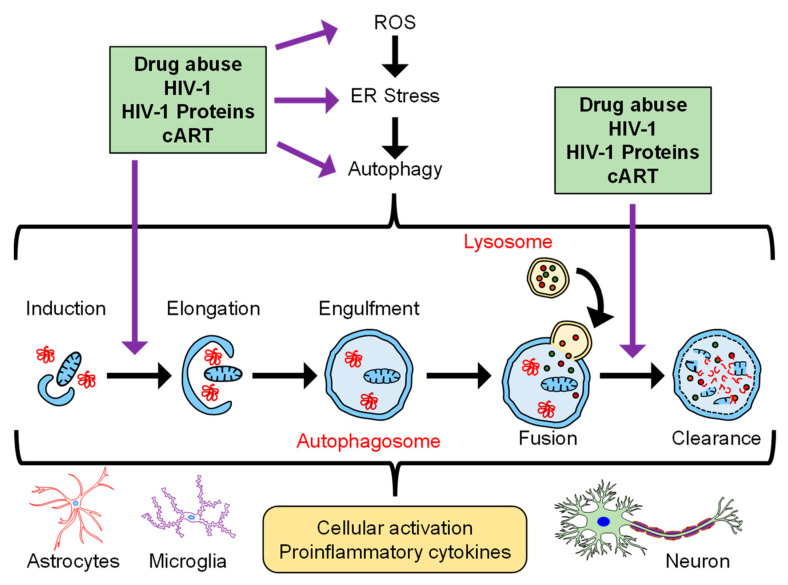
Schematic depicting the interplay of drugs of abuse, HIV-1/HIV-1 proteins, and cART in the context of autophagy and neuroinflammation.

Autophagy is made operational in a cell by fasting (deprivation of energy to cell), which makes the autophagosome release the engulfed components to provide whatever they have for the survival of cells in these deprived conditions [46]. This process of autophagy is highly coordinated and demands a fine-tuning balance when operational inside the cell, as any dysregulation in it causes cancer, infectious diseases, accelerated aging, and neurodegenerative disorders [47,48,49]. Figure 1 describes the interplay among drug abuse, HIV-1/HIV-1 proteins, and cART in the context of autophagy, cellular activation, and neuroinflammation.

### 2.1. Role of Autophagy in HIV and Drug Abuse

Autophagy has a profound role in maintaining nutrient levels inside the cells and in infectious diseases like HIV-1. HIV-1 cross-talks with autophagy in that the viral envelope causes autophagy-dependent cell death by binding to the C-X-C chemokine receptor type 4 (CXCR-4) on T cells [50]. Additionally, autophagy assists HIV-1 replication by playing a role in CD4+ T cells and macrophages and its involvement in HIV-1 pathogenesis [51]. There is repression of autophagy in CD4+ T cells infected with HIV-1; however, autophagy is highly activated in infected monocytes/macrophages [51]. Autophagy has been shown to counter the effect of HIV-1 Tat in CD4+ T lymphocytes by causing the degradation of HIV-1 viral particles [52]. HIV-1 has evolved to avoid autophagy-mediated degradation, for which many reasons remain unknown. One of the reasons this occurs is that the HIV-1 Nef interacts with Beclin-1, which in turn blocks the maturation of monocytes/macrophages to cause autophagy, and the HIV-1 avoids getting degraded [53]. Another mechanism the HIV-1 utilizes to evade the autophagy-mediated degradation is activating the mammalian target of rapamycin (MTOR) in dendritic cells, making CD4+ T cells more likely to become infected with the HIV-1. Both mechanisms mentioned above used by HIV-1 could be potential therapeutic targets for drug discoveries, and many other possible means of HIV-1-camouflaging cellular autophagy could be discovered in the future [54]. Drug abuse involving methamphetamine, cocaine, morphine, and alcohol has been reported to compromise the immune system, putting abusers significantly at higher risk of HIV-1 infection [55]. In addition, abusers are highly likely to acquire an HIV-1 infection because their mental illness acts as a precursor for aberrant behaviors, including unprotected sex and needle sharing, which increases the risk of getting HIV-1. There is an increase in autophagy in drug abuse subjects, confirmed by the presence of multi-membrane vacuoles in various cell types [56], which is a hallmark of autophagy. Hence, autophagy, HIV-1 infection, and drug abuse seem to go hand in hand.

### 2.2. Role of Inflammation in HIV-1 and Drug Abuse

During systemic inflammation, an increase in C-reactive protein (CRP) and serum amyloid A (SAA) are two critical indicators of inflammation known as acute phase proteins. The crucial link between inflammation, HIV-1, and drug abuse is that CRP and SAA affect inflammatory cytokines and immune dysfunction (which trigger HIV-1-associated neuropathogenesis progression) [57,58,59]. The acute-phase protein CRP binds strongly to phosphocholine residues and causes a shift in plasma lipoproteins constituents [60]. On the other hand, SAA induces the proinflammatory cytokines tumor necrosis factor-α (TNFα), interleukin (IL) 6, and IL4 [61]. Additionally, reports have shown that increased CRP and SAA in HIV-1 infection contribute to immune suppression and progression [62,63,64]. Furthermore, it has also been reported that drugs of abuse (methamphetamine, cocaine, alcohol) and HIV-1 infection have an increased risk of developing chronic neuroinflammation, enhanced immune dysfunction (predisposition to HIV-1 infection), and cognitive impairment [65,66,67,68,69,70]. This has been demonstrated in studies on various CNS cells (neurons, glial cells, and pericytes) exposed to HIV-1/HIV-1 proteins and drugs of abuse, which led to increased expression of proinflammatory cytokines (such as TNFα, IL1β, and IL6) in both the intracellular and extracellular compartments [24,25,30,38,65,71,72,73]. Additionally, it has also been reported that exposure of neurons and glia to HIV-1 proteins and drugs of abuse, such as cocaine, resulted in dysregulated autophagy, which was characterized by increased formation of autophagosomes, the defective fusion of the autophagosome with the lysosomes as well as defective auto/mitophagy [24,25,30,38,65,71,72,73]. Notably, the ability of the CNS cells to mediate crosstalk involving the extracellular vesicles also promotes progressive neuroinflammation and neuronal aging in the context of HIV-1 and drugs of abuse [74,75,76,77,78,79]. Cellular crosstalk propagating neuroinflammation is also mediated by proinflammatory cytokines released by the donor cells, which, in turn, can impact the functioning of the recipient cells [76,77,78,79,80]. 

### 2.3. Role of Aging in HIV-1 and Drug Abuse

Aging has been associated with drug abuse, and PLWH has further high usage of taking substances of abuse, including alcohol, tobacco, methamphetamine, and other drugs [81]. Aged subjects’ health is impacted by comorbid conditions as the drugs they abuse could easily interact with other medications they use for survival and cause loss of effectiveness of the medication, including cART in aged HIV-1 patients [82]. In addition, drug abuse in aged HIV-1 subjects demonstrates a significantly lower ability to cause viral suppression (ability to combat viral infection), leading to further aggravation of poor health, which becomes one of the causes of their mortality [83]. Therefore, an essential connection among aging, HIV-1, and drug abuse is that advanced age increases susceptibility to HAND in PLWH, who, later in their cognitive impairment, tend to use drugs more and therefore lose capacity to fight secondary infections [84]. It has been also reported that aged people who are active in sex, perform different (anal) sexual practices, and have unprotected sex, puts them in a high-risk group of contracting an HIV-1 infection [85]. What further aggravates it is that these active, aged, sex subjects are also users of abusive drugs, which significantly complicates aging.

## 3. Microglia, Autophagy, and Aging

Microglia and brain resident macrophages play a significant role in the innate immune system of the CNS and orchestrate inflammatory responses in the brain due to damage in the CNS. Microglia regulate the release of both pro- or anti-inflammatory cytokines and are involved in the phagocytosis of debris in the CNS [86,87]. Microglia are also known to play diverse roles in maintaining brain health and functions, including pruning of synaptic structures, remodeling of the perisynaptic environment, neuronal plasticity, modulation of neurogenesis, and the release of neurotropic soluble factors, as well as cytokines in the brain [88,89,90]. Based on the nature of the activating stimulus, the microglia respond differently, and release cytokines and chemokines related to either pro- or anti-inflammation. Activation of microglia to the “classic M1 polarization” releases proinflammatory mediators and contributes to neuronal and other glial cell damage. In contrast, the activation of the alternative M2 polarization limits inflammation by yielding anti-inflammatory factors and supports neurons via secreting neuronal growth factors [87,91,92]. The activation of proinflammatory response-as well as chronic altered neuroinflammatory status, including changes in microglial morphology, phenotype, and activity-have been implicated as a potential mechanism of neurodegeneration and progression of age-associated neurodegenerative disease, including Parkinson’s disease (PD), Alzheimer’s disease (AD), and amyotrophic lateral sclerosis (ALS) [93,94,95,96]. Several lines of evidence have confirmed that the autophagy pathway regulates innate and adaptive responses and the outcome of inflammatory reactions, phagocytosis, and antigen presentation in macrophages and microglia [97,98,99]. Intriguingly, it has been documented that impaired autophagy flux in macrophages is well associated with low-grade chronic inflammation and immune senescence, known characteristic features of aging [100,101,102].

Microglia activation-mediated low-grade chronic inflammation has been known to be associated with the progression of “inflammaging” in the CNS and the peripheral tissues [101,103,104,105]. Intriguingly, microglia derived from the brains of aged mice displayed increased basal levels of proinflammatory cytokines, including TNFα, IL1β, and IL6 [106]. Furthermore, studies from different models, including humans, rodents, canines, and non-human primates, showed age-associated microglial priming and an increased inflammatory profile of the CNS, such as major histocompatibility complex II, the cluster of differentiation molecule (CD) 11b, scavenger receptor CD68, CD11c integrins, TLRs and co-stimulatory molecule CD86 in the aged brain [95,107,108,109,110,111,112,113]. Intriguingly, the microglia in aged brain dogs, gerbils, and mice showed an activated morphology of a de-ramified structure, along with an increased ionized calcium-binding adaptor protein-1 (Iba1), a protein expressed on the surface of microglia in young adults [114,115]. Autophagy dysfunction was significantly associated with microglial activation and neuroinflammation [38,92,116,117], which contributed to neuronal damage and the progression of age-related neurodegenerative diseases [98,103,118]. A recent study reported that modulation of the autophagy pathway regulates the polarization of microglia [92]. TNFα exposure directed the microglia towards the M1 phenotype, as evidenced by increased M1 marker (inducible nitric oxide synthase [iNOS], IL1β, and IL6) expression and the reduction of M2 marker (Arginase1, and IL10), which is associated with impaired autophagy flux. It has been documented that those microglia exposed to TNFα promoted the microglia polarization towards M2 upon upregulation of autophagy, either via serum deprivation or pharmacological activators, such as rapamycin and resveratrol. On the other hand, pharmacological or genetic autophagy inhibitors using 3-methyladenine (3-MA) or autophagy-related (ATG) 5 siRNA consistently aggravated the M1 polarization in microglia challenged with TNFα [92]. Recent studies from our group have confirmed that either cocaine or HIV-1 Tat stimulates microglial activation and neuroinflammation due to increased mitochondrial dysfunction (along with lysosomal dysfunction), which results in defective clearance of damaged mitochondria via the mitophagy (selective mitochondrial autophagy) pathway [38,117]. Another study from our group also documented that an antiretroviral drug cause increased microglial activation via increased permeabilization of lysosomes and impaired autophagy flux [119]. A recent study also showed that lipopolysaccharide (LPS)-induced neuroinflammation in N9 microglial cells associated with the inhibition of autophagic flux through activating the PI3KI/AKT/MTOR pathway; however, enhanced microglial autophagy downregulates LPS-induced neuroinflammation [120]. 

Apart from an inflammatory response, microglia also phagocytize the different types of brain-derived cargo, including neuronal and myelin debris, apoptotic cells, protein deposits, and other substrates. Autophagy plays a significant role in the clearance of those engulfed cargo via lysosomal fusion and degradation [86,98,121]. Studies have shown that increased accumulation of misplaced or altered molecules, resulting from damaged and dead cells and organelles (cell debris) due to defective auto/mitophagy, could activate receptors of an innate immune system and neuroinflammation, in turn accelerating the aging process [122,123,124,125]. A recent study reported that microglia-mediated protection of neurons involved synucleinphagy, where the α-synuclein released from neurons was removed by microglia. Synucleinphagy is mediated via TLR4/NF-κB/p62 signaling pathway in microglial cells of mice expressing human α-synuclein in the neurons [126]. Another study by Pomilio et al. [127] documented that prolonged or chronic exposure of microglia to β-amyloid (Aβ) peptides resulted in impaired autophagy in microglial cells both in vitro and in vivo, as well as in AD patients, in turn, contributing to the progression of the disease. It has also been reported that increased mitophagy using nicotinamide adenine dinucleotide supplementation diminished expression of insoluble Aβ1–42 and Aβ1–40 forms via increased microglial phagocytosis of extracellular Aβ plaques, in turn, ameliorating neuroinflammation and cognitive impairment in an APP/PS1 mouse model. Furthermore, this study also documented that the induction of mitophagy and reversal of cognitive impairment involved the PTEN-induced kinase-1 (PINK-1) and parkin-dependent pathways [128]. There is also evidence that alleviating impaired autophagy flux via pharmacological or genetic approaches prevented microglia activation and neuroinflammation [38,71,116,117,129,130], ultimately preventing neuroinflammation. 

## 4. Astrocytes, Autophagy, and Aging

Astrocytes are the most abundant CNS cell type and have a multitude of functions in the brain, including maintenance of cellular homeostasis. Additionally, autophagy in astrocytes is believed to play a vital role in the pathogenesis of aging and neurodegenerative diseases. Several reports have shown that autophagy can influence inflammation, oxidative stress, and the aging process in different cell types, and interrelation among the different factors have been well-studied, but in relation to astrocytes are small. It has been reported that activation of astrocytes is an early event in PLWH [131] and in animal models of NeuroHIV [132]. Earlier studies showed that activation of the autophagic signaling pathway was required for HIV-1 replication, and an inhibition in this pathway resulted in attenuation of viral replication [133,134]. This autophagy dysregulation is considered essential for HIV-1 replication and can also be associated with HAND [135]. HIV-1 proteins have also been shown to regulate astrocytic functions via direct modulation of autophagy pathways [136]. For instance, HIV-1 gp120 has been shown to play a pro-survival role against astrocyte cell death via induction of the autophagy pathway [136]. It has also been observed that HIV-1 Tat can induce autophagy by upregulation of a Bcl-2-associated athanogene 3 (BAG3) and NF-κB signaling pathway [137]. Abundant expression of the HIV-1 viral protein Nef was observed in HIV-1-infected astrocytes [138], and this Nef protein was shown to induce neurocognitive impairments in rats [139,140]. Overexpression of Nef in human astrocytes resulted in the expression of key molecules of the autophagy pathway, e.g., ATG8, LC3II, and p62 [138]. Exposure of astrocytes with autophagy inhibitors resulted in significant release of Nef in the extracellular vesicles (EVs) derived from primary human fetal astrocytes, which, upon being uptaken by the neurons, resulted in inhibition of neuronal action potential and therefore induced neurotoxicity [141]. Some other reports have shown that HIV-1 suppresses autophagy by disrupting the conversion of LC-I to LC3-II and induces toxicity in astrocytes [134]. Another study has shown that mitophagy is crucial for cell death resistance in HIV-1 productively infected astrocytes, but its impairment may favor inflammasome-mediated cell death in abortively infected cells [142]. These studies show the importance of the autophagic pathway in astrocytes with HIV-1 or viral proteins.

Drugs of abuse, such as opiates, can potentiate HIV-1 infectivity. Opioids have been shown to enhance HIV-1 replication and interact with opioid receptors in astrocytes, inducing the release of astrocyte-derived proinflammatory factors and potentiate neurodegeneration in HIV-1 patients on opiates [143,144], and can result in cognitive impairment [145]. Drugs of abuse, such as cocaine and methamphetamine, have also been implicated in potentiating HIV-1-induced neuropathogenesis [146,147,148]. Periyasamy et al. reported that cocaine-mediated dysregulated autophagy led to astrocytosis, which resulted in increased expression of proinflammatory mediators, such as TNF, IL1β, and IL6, leading to neuroinflammation [25]. Another study has shown that cocaine-induced autophagy led to type II programmed cell death in astrocytes [149]. Methamphetamine was also shown to induce autophagy in astrocytes via the opioid receptor and metabotropic glutamate receptor subtype 5 receptor-mediated signaling, whereas inhibition of autophagy exacerbated methamphetamine-induced apoptotic cell death [136]. Co-treatment of astrocytes with HIV-1 and morphine resulted in increased expression of p62, indicating blockage in the fusion of autophagosomes with lysosomes, resulting in the induction of inflammation [150]. Studies by Sil et al. demonstrated that morphine exposure to human astrocytes results in the induction of an ER stress-induced autophagy pathway, which leads to astrocytosis and neuroinflammation [151]. All these studies showed that HIV-1 alone, or in combination with different drugs of abuse, can induce autophagy-induced neuroinflammation in astrocytes, which can contribute to the process of inflammaging.

Aging is characterized by loss of regenerative capacity (which leads to morbid conditions) and has been considered a comorbid condition in HIV-1 and substance abuse disorders [4,5]. Studies have shown that structural and metabolic parameters of the brain decline at relatively younger ages in HIV-1-infected apolipoprotein E4 (ApoE4) carriers, leading to cognitive deficit and inflammation [152,153]. However, neuropsychological studies on HIV-1-infected cohorts have produced conflicting results regarding the impact of ApoE4 or the Apoε4 allele on the course of cognition. During normal aging, ApoE4 contributes to cognitive impairment by transcriptional repression for some genes related to cognitive reserve, and the same effect is observed in aging HIV-1 populations [154,155]. Studies on serum from HIV-1-infected patients have demonstrated the association between inflammatory markers, such as C-X-C motif chemokine ligand 1 and transforming growth factor (TGF) α, and increased telomere length (indicative of aging). Furthermore, IL-10RA on neurons was associated with decreased telomere length [156], thus indicating inflammaging. Studies have shown that antiretroviral therapy induces cell cycle arrest, senescence-associated beta-galactosidase, and the cell cycle inhibitor p21 in astrocytes. Further studies have also shown that anti-retroviral-induced astrocytic senescence can be associated with oxidative stress and metabolic changes that could play a role in the development of HAND [157].

Several drugs of abuse have been shown to potentiate HIV-1 infectivity and inflammation via the autophagy pathway, so their role in HIV-1-induced aging will be essential to be considered. It was reported that self-administration of cocaine reduced the uptake of neuron-derived EVs (NDEVs) by the astrocytes, primarily in the motor cortex, which can be effectively reversed by extinction training [158]. One study demonstrated that HIV-1 and methamphetamine induce senescence of primary human astrocytes, as evaluated by induction of senescence markers (β-galactosidase and p16), senescence-associated morphologic changes, as well as cell cycle arrest via the β-catenin pathway. HIV-1 and methamphetamine-mediated astrocyte senescence was also demonstrated in three small animal models (the humanized mouse model of HIV/NSG-huPBMCs, HIV-transgenic rats, and in a methamphetamine administration rat model). Senescent astrocytes, in turn, caused neuronal toxicity. Thus, the β-catenin pathway can be a potential therapeutic target to overcome astrocyte senescence [159]. Drugs of abuse like cocaine, morphine, and methamphetamine can induce inflammation as a result of the constant and consistent release of proinflammatory cytokines [25,151,160,161]. They can also potentiate inflammatory status in HIV-1 conditions [162,163], which can contribute to the process of inflammaging and will be an essential aspect to study in addicted HIV-1 patients undergoing premature aging. Autophagy has been closely associated with aging, i.e., the autophagic pathway was shown to decline during aging [164]. Inhibition of the target of rapamycin complex 1 (TORC1), which is a known negative regulator of autophagy, enhances lifespan [165]. The exercise was shown to downregulate TORC1 activity and upregulation of autophagy in different tissues and thus may represent practical means to promote longevity [165]. Recently, it was reported that there were reductions in autophagic flux in C. elegans, which corroborates with organismal lifetime since there was an age-dependent accumulation of autophagosomes, thus decreasing autophagic degradation [166]. Therefore, autophagy is linked with aging. There are scanty reports on HIV-1 and drug-mediated autophagy linked to aging, but further investigation on the role of autophagy in aging will open promising avenues for anti-aging drug development.

## 5. Neurons, Autophagy, and Aging

Aging leads to neuronal alterations–such as changes in neuronal morphology, neuronal damage, and dysregulation of neuronal signaling pathways–which also contribute to neuroinflammation and aging. Emerging studies have demonstrated that neuronal autophagy plays an essential role in neuroinflammation, aging, and HIV-1 dementia [167,168,169]. Atg7 is an E1-like enzyme required for autophagosome biogenesis [170]. To understand the role of neuronal autophagy in aging and age-related disease, Rudnick et al. generated motor neuron-specific autophagy-deficient mice by crossing Atg7^flox/flox^ mice to ChAT-Cre mice (Atg7 cKO) [171]. Interestingly, inhibition of motor neuron autophagy significantly extends lifespan in Atg7 cKO mice compared with Atg7 cWT mice [171]. A study from the same group demonstrated that TANK-Binding Kinase 1 (TBK1) is active in ubiquitinated inclusions in motor neurons and is directly involved in the autophagy pathway that targets protein aggregates for clearance [116]. These findings thus indicate that autophagy plays both beneficial and detrimental roles in aging and age-related diseases.

In transgenic mice expressing exon 1 of the human HD (Huntington’s disease) gene carrying a CAG repeat expansion, Petersén et al. demonstrated that the mutant striatal neurons exhibited elevated cell death and increased autophagic granules and electron-dense lysosomes in response to dopamine treatment compared with wild-type neurons [172]. These findings suggest that the combinatorial effect of mutant neurons in the Huntington gene and dopamine-induced oxyradical stress promotes autophagy, which could, in turn, lead to cell death underlying age-related diseases. In line with these findings, Purkinje neuron death has also been characterized by excessive autophagic-lysosomal vacuolation [173]. Florez-McClure et al. demonstrated that trophic factor withdrawal induces a marked increase in the sizes of autophagic and lysosomal vacuoles, resulting in a nonapoptotic death pathway in Purkinje neurons [173]. Moreover, antisense oligonucleotides to the p75 neurotrophin receptor (p75ntr) decrease autophagy and inhibit trophic factor withdrawal-induced Purkinje neuron death [173]. The authors further demonstrated that the p75ntr supports Purkinje neuron survival in the presence of trophic factors but promotes autophagy and death of Purkinje neurons in the absence of trophic stimuli [173]. Emerging studies suggest that HIV^+ve^/HD patients exhibit an earlier onset of HD symptoms than HIV^−ve^/HD patients [174,175,176]. Studies also demonstrated that in these patients, dysregulation of neuronal autophagy was associated with HIV-1 dementia and encephalitis [167,168]. As the brain is a target of various drugs of abuse, including morphine, which results in various cellular signaling alterations [177,178], it is not surprising that increased neuronal loss was found in HIV-1 Tat and morphine-administered *Becn1^+/+^* mice but not in *Becn1*^+/−^ mice [169,179]. These findings also suggest a role of autophagy in HIV-1 Tat and morphine-mediated neuronal damage. However, whether and how neuronal autophagy is involved in the earlier onset of HD in HIV^+ve^/HD patients on drugs warrants further investigation.

High-mobility group box 1 (HMGB1) is a nuclear protein and can be released into the extracellular space to act as an inflammatory mediator [180,181]. Zhu et al. demonstrated that extracellular HMGB1 induces autophagy in primary spinal neurons underlying spinal root avulsion [182]. This was further demonstrated that HMGB1-induced autophagy increases the survival of primary spinal neurons under oxygen-glucose deprivation conditions [182]. These findings suggest a beneficial role of neuronal autophagy. However, in another study, Yin et al. demonstrated that inhibiting autophagy by salidroside protected neonatal neurons from glutamate-induced apoptotic cell death [183]. A study by Tripathi et al. also showed that impairment of autophagy in human motor neurons induced by reactive astrocytes secreted TGF-β1 and led to ALS-like axonal and cytoplasmic protein aggregation in the neurons [184]. Similarly, Lang et al. found that dopaminergic neurons significantly enhanced autophagy in PD mice [185]. The authors further demonstrated that the upregulation of long non-coding RNA HOTAIR promoted autophagy in mid-brain dopaminergic neurons of the substantia nigra compacta in PD mice [185].

To examine whether motor neuron autophagy plays a role in amyotrophic lateral sclerosis (ALS), Tian et al. generated a double transgenic (DTg) mouse model by crossing green fluorescent protein (GFP)-fused LC3 transgenic (LC3-Tg) mice with G93A mutant human Cu/Zn superoxide dismutase (mSOD1) transgenic (mSOD1-Tg) mice [186]. Imaging analysis revealed a solid increased autophagic signal in both the spinal motor neurons and surrounding glial cells [186]. The authors also found that the number of neurons displaying autophagy in the DTg mice reduced at the end symptomatic stage, with decreased numbers of living motor neurons [186]. These results suggest that autophagy-mediated motor neuronal death could be involved in the progress of ALS.

Hypothalamic autophagy reduces during aging [187]. Loss of autophagy in hypothalamic proopiomelanocortin (POMC) neurons have been shown to impair the generation of α melanocyte-stimulating hormone (α-MSH) and lipolysis [188]. These data suggest that POMC neuronal autophagy plays an essential role in maintaining energy balance, whereas reduced autophagy in POMC neurons could lead to metabolic disturbances observed during aging [188]. Mice with specific deletion of autophagy-related 7 (Atg7), an essential autophagy gene, in hypothalamic POMC neurons (Atg7ΔPOMC mice) exhibited increased food intake and decreased energy expenditure with a concomitant increase in body weight [189]. These findings thus demonstrated the critical role of autophagy in POMC neurons in controlling appetite balance and energy homeostasis [189]. Increasing evidence suggests that autophagy plays a vital role in neuroinflammaging. Interestingly, studies have revealed diverse roles for autophagy in neurons (depending on the type of neurons), the status of the disease, and the stage of age. Therefore, outcomes of investigation on neuronal autophagy could pave the way for the future development of neuronal autophagy-targeted strategies in the prevention and treatment of neuroinflammaging.

## 6. The Role of Autophagy in Aging-Associated Blood–Brain barrier (BBB) Disruption

BBB is a tight barrier critical for preventing pathogens and small molecules from entering the brain. It is composed of endothelial cells, pericytes, and end-feet of astrocytes [190]. Disruption of the BBB contributes to the pathogenesis of many CNS diseases, including PD [191], AD [192], HAND [193], etc. Moreover, the breakdown of BBB was considered an early hallmark in the aging brain [190] and recognized as an early biomarker of cognitive impairment in AD [194]. As conveyed in previous sections, autophagy plays a crucial role in cellular/organismic aging, age-related dysfunction, and disease. Impairment of vascular autophagy could contribute to the development of oxidative stress, inflammation, and vascular dysfunction with aging through the loss of ability to remove dysfunctional proteins/organelles and to recycle damaged biomolecules that interfere with normal cellular function. Although evidence suggests that autophagy declines with aging in numerous tissues [195,196], its contribution to the development of vascular dysfunction with aging, mechanisms involved, and aging-associated neurological disease are largely unknown. We will provide a brief overview of previous findings on the role of autophagy underlying age-associated BBB dysfunction.

### 6.1. Endothelial Cells

The tight junction (TJ) complexes are molecules that interact in the extracellular junctional space and act as anchors between endothelial cells [197], maintaining the barrier functions of BBB. Tight junctions are integral membrane proteins, including claudins, occludin, junctional adhesion molecules (JAM)-A, -B, -C, and scaffolding proteins like zonula occludens (ZO-1, ZO-2, and ZO-3) [198]. During aging, it has been demonstrated that the expression of BBB tight junction proteins, occludin-1, and ZO-1, were significantly attenuated in aged mice compared to young mice [199]. Additionally, the number of tight junctions decreasing with advanced age was also reported in rats, resulting in increased BBB permeability [200]. The permeability of BBB is closely associated with the expression levels of these tight junction proteins. The function of tight junctions’ regulating permeability is based on two pathways: (1) the “pore” pathway that can carry small uncharged solutes and specific ions and (2) the “leak” pathway that can carry non-charged larger molecules [201]. The TJ membrane proteins, such as claudins (such as claudin-2), have been shown to play a crucial role in regulating the cation-selective pore pathway, while the barrier-forming protein occludin is known to regulate the leak pathway. Induction of autophagy has been shown to result in the degradation of claudin-2, leading to the enhancement of the TJ barrier function with increased transepithelial resistance. In a recent study, exposure of primary human brain microvascular endothelial cells to HIV-1 Tat-induced colocalization of LC3B puncta with ZO-1 and HIV-1 Tat-mediated downregulation of ZO-1 was attenuated by pretreating the cells with pharmacological inhibitors of autophagy, as well as by silencing ATG5. Collectively, these findings underpin the role of autophagy in the HIV-1 Tat-mediated downregulation of ZO-1, which, in turn, leads to increased permeability of BBB [202].

### 6.2. Pericytes

Pericytes, essential constituents of the cerebrovascular unit, play a crucial role in maintaining the integrity of the blood–brain barrier [203]. Several studies have demonstrated pericyte coverage is positively correlated with the integrity of BBB [204] and modulating the influx of immune cells [205,206], controlling blood flow, etc. [203]. Emerging studies have suggested that pericytes may control critical neurovascular functions, and pericyte loss may play a role in age-dependent vascular-mediated neurodegeneration [205]. Additionally, pericytes were found to regulate capillary blood flow in the aging mouse brain, which might be the mechanism underlying aging-associated neurovascular dysfunction [207]. However, the mechanism by which age-dependent pericyte dysfunction regulates blood flow and the mechanisms of aging-associated pericyte loss is still mostly unclear. Recently, Yuan Zhang et al. reported that the knockdown of the sigma-1 receptor (σ-1R) resulted in pericyte loss via increasing autophagy and apoptosis, which, in turn, led to BBB disruption and thus contributed to cerebral infarction [208]. Furthermore, they found YZ001 (σ-1R agonist) -alleviated pericyte loss via inhibition of autophagy, resulting from σ-1R activation, indicating a novel role of σ-1R in mediating pericyte survival through regulating the interplay between apoptosis and autophagy. While pericytes have also been reported to be productively infected with HIV-1 [209], studies are warranted to investigate the role of these cells in regulating autophagy and aging in the context of BBB disruption. Overall, future studies are needed to explore the involvement of the autophagy pathway in aging-mediated pericyte disruption, which could provide a possible therapeutic strategy for aging-associated neurological diseases.

## 7. Conclusions

Overall, defective or blocked autophagy is an essential target in activating CNS cells such as microglia, astrocytes, and neurons, leading to the continued release of proinflammatory cytokines. Various reports implicate defective autophagy as an upstream signaling pathway in the generation of the inflammatory response, leading to neuroinflammation and neuroinflammaging. Interestingly, membrane phagophore emerge in the first place when specific cellular cargo is targeted for degradation, and alternatively, independent Atg5/Atg7 mechanisms are regulated in the context of HIV-1, and drug abuse remains unknown. Interactions aimed at blocking pathways involved in HIV-1 and a drug abuse-mediated role in defective autophagy could be considered a future therapeutic strategy to dampen the effects of sustained inflammation caused by these CNS assaults. The use of rapamycin or hypoxia-inducible factor-dependent target genes that reduce mTOR activity, which leads to the induction of autophagy, ensures that cells adapt to their changing environment, eliminate toxic episodes, and promote cell viability, can be considered as a potential target for restoring cognitive deficits associated with neurodegenerative disorders and neuroinflammaging. Thus, autophagy could emerge as a potent modulator of neuroinflammaging in HIV-1 and drug abuse-mediated pathologies.

## Data Availability

Not applicable.
